# Mechanisms regulating intestinal barrier integrity and its pathological implications

**DOI:** 10.1038/s12276-018-0126-x

**Published:** 2018-08-16

**Authors:** Chaithanya Chelakkot, Jaewang Ghim, Sung Ho Ryu

**Affiliations:** 1 Division of Integrative Biosciences and Biotechnology, Pohang University of Science and Technology, Pohang, Republic of Korea; 2 NovaCell Technology, Inc., 77 Cheongamro, Pohang, Gyeongsanbuk-do Republic of Korea; 3 Department of Life Sciences, Pohang University of Science and Technology (POSTECH), Pohang, Republic of Korea

## Abstract

The gastrointestinal tract is a specialized organ in which dynamic interactions between host cells and the complex environment occur in addition to food digestion. Together with the chemical barrier of the mucosal layer and the cellular immune system, the epithelial cell layer performs a pivotal role as the first physical barrier against external factors and maintains a symbiotic relationship with commensal bacteria. The tight junction proteins, including occludin, claudins, and zonula occludens, are crucial for the maintenance of epithelial barrier integrity. To allow the transport of essential molecules and restrict harmful substances, the intracellular signaling transduction system and a number of extracellular stimuli such as cytokines, small GTPases, and post-translational modifications dynamically modulate the tight junction protein complexes. An imbalance in these regulations leads to compromised barrier integrity and is linked with pathological conditions. Despite the obscurity of the causal relationship, the loss of barrier integrity is considered to contribute to inflammatory bowel disease, obesity, and metabolic disorders. The elucidation of the role of diseases in barrier integrity and the underlying regulatory mechanisms have improved our understanding of the intestinal barrier to allow the development of novel and potent therapeutic approaches.

## Gut health: intestinal barrier function underpins health and disease

A better understanding of how the cells that line the inside of the intestines allow nutrients in, while keeping harmful substances and pathogens out could lead to new therapies for inflammatory bowel disease, obesity, and other conditions. A team from South Korea led by Sung Ho Ryu from Pohang University of Science and Technology review the regulatory mechanisms that help maintain the intestinal epithelial barrier. They discuss the role of tight junction proteins in forming a seal between adjacent cells and the various signaling pathways that loosen or tighten these junctions to enable limited transport. Loss of barrier integrity because of genetics, gut microbes, auto-immunity, diet, or other factors is often implicated in disease, and restoring barrier function with drugs or probiotics could help ameliorate many health problems.

## Introduction

The intestinal epithelial layer forms the major barrier that separates our body from the external environment. Trillions of commensal bacteria reside in the gastrointestinal tract and have a vital role in digestion and the development of the immune system. However, they present a risk of infection^1^. The maintenance of the intestinal epithelial barrier is the essential function of the intestinal epithelial cells (IECs). The IECs integrate positive and negative interactions from the microbiota living in the gut and signal the immune cells to accommodate the microbiota, thereby perpetuating the normal function of the body^2–4^. An imbalance in the intestinal barrier structure can flare up into an uncontrollable immune reaction in the intestinal microenvironment or allow the unrestrained growth of microbiota, which leads to various diseases, including intestinal inflammatory disorders, extra-intestinal autoimmune diseases such as rheumatoid arthritis and multiple sclerosis, and metabolic disorders such as diabetes and obesity^4–6^. Critically ill patients and patients receiving chemotherapy/radiotherapy show severely compromised intestinal barrier integrity. Asymptomatic close relatives of patients with inflammatory bowel diseases (IBDs) also show compromised barrier integrity. Intestinal barrier permeability may therefore be a prognostic marker for disease pathophysiology; similarly, targeting the intestinal barrier permeability holds promise for therapy and for the prevention of disease.

## Intestinal epithelial barrier

**Fig. 1:**
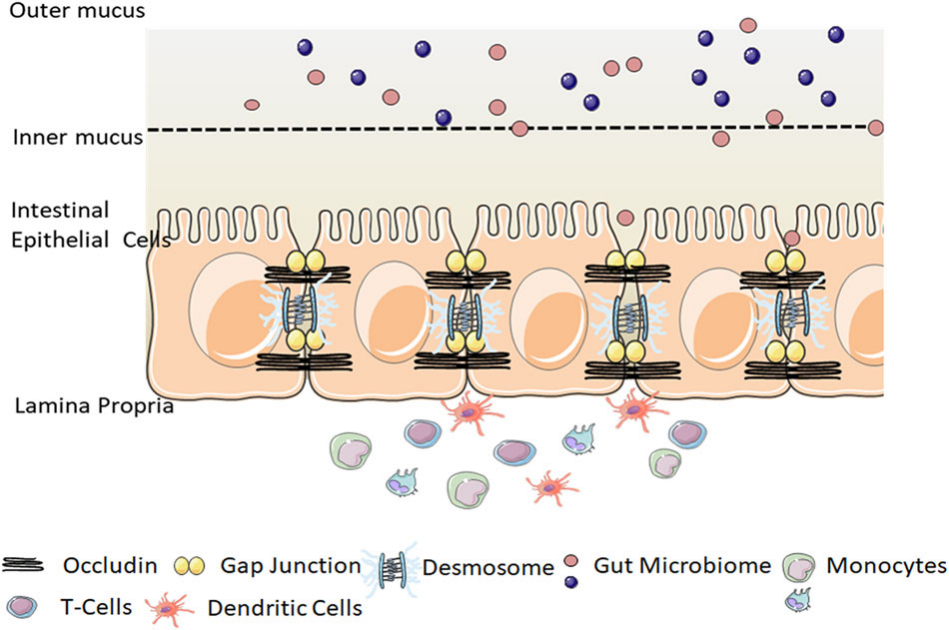
Intestinal epithelial barrier. Epithelial cells form a layer that functions as a physical barrier facilitated by tight connections between each cell. A number of tight junction protein components seal the paracellular pathway and conduct gate and fence functions. The mucosal layer is a chemical barrier that is critical to limit the contact between the microbiome and epithelial cells. Immune cells are also a major participant in the immune response and the tolerance of the host against external substances. The graphical illustration was drawn by using the images from Servier Medical Art by Servier, with slight modifications (http://www.servier.com/Powerpoint-image-bank, https://creativecommons.org/licenses/by/3.0/)

The intestinal epithelial barrier is a one-cell-thick internal lining of the gut that contains different types of epithelial cells. Underneath the epithelial layer, there is a thin layer of connective tissue, the lamina propria, which has a crucial role in nurturing healthy communication between the microbiome and the immune cells. The intestinal epithelial system is also home to immune cells, including dendritic cells, T cells, B cells, and macrophages, which function in close relation with the IECs to maintain intestinal homeostasis^3,7^. Gut microbiota, consisting of hundreds of trillions of bacteria and viruses, are pivotal for the maintenance of a symbiotic relationship with immune cells^8–10^. Recent studies have reported compelling evidence for the metabolic, immunological, and physiological roles had by the gut microbiota. However, the first layer of defense in the epithelium of the gut is formed by a layer of mucus, which is critical for the limitation of the exposure of epithelial cells to the microbiome^11^. The absence of mucin, a highly glycosylated polymeric protein in the mucous layer, makes an animal vulnerable to intestinal inflammation, which leads to the development of spontaneous colitis and confers a predisposition to the development of colorectal cancers (Fig. 1).

## Cellular functions of IECs

The intestinal epithelial layer is highly dynamic and characterized by a remarkable turnover rate; IECs are rapidly renewed and replaced every couple of days^12,13^. The maintenance of this cell layer renewal requires tight regulation to avoid any imbalance in homeostasis^14,15^. The intestinal epithelial monolayer is composed of different types of specialized epithelial cells, such as enterocytes, Paneth cells, goblet cells, endocytes, and microfold cells, each with a distinct function. The most abundant of these are IECs or enterocytes, for which the major function is the maintenance of epithelial barrier integrity^16,17^. Paneth cells reside in the base of crypts and secrete anti-microbial peptides, such as α-defensin, to impede microbial entry to the intestinal lumen. Goblet cells secrete mucous, trefoil peptides, and resistin-like molecule-β, which are central to both the defense and repair of the epithelial layer and have significant roles in epithelial homeostasis^18–20^. Endocytes regulate incoming antigens and microfold cells secrete IgA, which, in addition to goblet cells, helps present bacterial antigens to dendritic cells. Collectively, these cells form a polarized layer to establish a tight barrier by virtue of intracellular tight junctions, adherens junctions, and desmosomes.

IECs are capable of phagocytosing bacteria and can also sequester and neutralize bacterial toxins. These cells are also specialized to recognize bacterial-derived molecules, known as prokaryotic-associated molecular patterns, with the help of the Toll-like receptors on the cell surface and the nucleotide-binding oligomerization domain-like receptors in the cytoplasm, which activate defense mechanisms by the secretion of anti-microbial peptides^2^. IECs also maintain two-way communication with the underlying immune cells to regulate the inflammatory response against bacterial toxins^21^. In conjunction with the mucosal layer and specialized cells, the epithelial layer forms a well-equipped, intricately regulated and stringent barrier with continuous scrutiny by immune cells to create an immune-silent environment.

## Mechanism of regulation of intestinal epithelial tight junction proteins

A crucial function of IECs is the maintenance of barrier integrity, which allows the permeability of essential ions, nutrients, and water but restricts the entry of bacterial toxins and pathogens^21^. The transport of molecules across the epithelial layer occurs through three major pathways: the trans-cellular pathway (passive diffusion across the cell membranes), the carrier-mediated pathway (carrier/receptor-mediated trans-cellular pathway), and the paracellular pathway (passive diffusion between the spaces through adjacent cells). The epithelial tight junction proteins, the most apical component of epithelial intracellular junctions, equip IECs with this function, which seals the paracellular space between the cells and tightly restricts the transport of hydrophilic molecules^22–24^. That is, the main function attributed to the tight junction proteins is the “gate and fence function,” which allows the paracellular transport of some solutes and molecules but prevents the intramembrane transport of proteins, lipids, and microbial-derived peptides^25,26^. Any alteration in the tight junction structure can prove to be detrimental to the organism.

The tight junction is composed of several transmembrane and cytosolic proteins, including occludin, claudins, zonula occludens (ZOs), tricellulin, cingulin, and junctional adhesion molecules (JAM), which interact with each other, as well as with the cytoskeleton, and form a complex architecture^27^. Most of these proteins, except for cingulin and ZO, are integral membrane proteins that extend into the paracellular spaces between the cells. Cingulin and ZOs are cytoskeletal linker proteins, which interact with the cytoplasmic peripheral membrane proteins, occludin, claudin, and JAM to form strong cross-links and interact with the membrane cytoskeleton composed of F-actin and myosin. Together with intracellular signaling proteins, tight junction proteins activate a plethora of cellular processes to maintain barrier integrity^28^. Tight junction complexes are the rate-limiting factor for paracellular permeability; they are programmed to rapidly open and seal the barrier in the event of injury and other signals. They form a highly dynamic entity, continuously transmitting signals to the individual components that undergo a series of regulations to enhance or modulate the integrity of the intestinal barrier.

Although it is well-accepted that tight junctions are crucial for the maintenance of barrier integrity, the exact function of the individual tight junction proteins remain elusive. Over the years, researchers have highlighted the diverse functions performed by tight junction proteins. Occludin, the first identified tight junction protein,^29,30^ has a dual role in the intestinal barrier; it provides structural integrity to the tight junction and is an integral component in the barrier function of tight junctions^31^. The expression level of occludin was found to be closely correlated with the barrier properties in vitro and in vivo^32–35^. Interestingly, occludin knockout mice had morphologically intact tight junctions but displayed complex histological phenotypes, with chronic inflammation and a defective epithelial barrier, which implicated that its crucial role was in tight junction stability rather than tight junction assembly. In contrast, certain other studies reported normal barrier function in occludin-deficient mice but showed chronic inflammation and hyperplasia in the gastric epithelium and testicular atrophy^36,37^. Severely compromised occludin expression has been observed in disease models of intestinal inflammatory diseases, which suggests it has a critical role in the maintenance of barrier integrity^38–40^. Collectively, these studies indicated that the functions of occludin are complex, and the mechanism by which occludin regulates the tight junction should be investigated in great detail.

Claudins, the other major tight junction proteins, are responsible for the regulation of paracellular space^27,41,42^. There are several isoforms of claudin, each having potentially different roles^43,44^, and a fine balance between them is needed for the maintenance of paracellular integrity. Alterations in the claudin levels can affect the intestinal barrier integrity in different ways depending on the type of claudin isoform^45^. For example, the downregulation of claudin 5 and 8 can drastically reduce the barrier integrity^46^; in contrast, claudin-2, a tight junction protein required for the formation of paracellular water channels that is highly expressed in leaky epithelial tissues, is upregulated in IBDs and promotes inflammation^27,46,47^. ZOs are peripheral membrane-associated proteins ubiquitously expressed in epithelial and endothelial cells. The various isoforms, ZO-1, ZO-2, and ZO-3, are all characterized by their ability to interact with different cellular proteins through a multitude of protein binding domains, such as the SH3 domain, the PDZ domain, and the leucine-zipper domain,^48,49^ and are also essential for scaffold formation and the connection of other tight junction proteins to the cytoskeleton. JAM-A, another tight junction protein, is also implicated in the maintenance of intestinal barrier integrity. JAM-A-deficient mice have increased barrier permeability with elevated bacterial translocation; however, they do not develop spontaneous colitis^50,51^. The roles of other tight junction proteins and their mechanism of action remain largely unknown.

Tight junction proteins are closely regulated, which is imperative for the maintenance of normal barrier integrity. IECs proliferate rapidly and renew quickly, and it is essential that the tight junction proteins are also strictly regulated to avoid any detrimental effect on membrane integrity^52^. They are also capable of efficiently adapting to the different demands of the cell by sealing, opening, and maintaining paracellular transport under various physiological and pathological conditions^53^.

The mechanism of the regulation of tight junction proteins is intricate and somewhat obscure. The tight junction proteins are regulated by multiple signaling proteins and signaling molecules. Several molecules involved in the signal transduction processes, including small GTP-binding proteins and tyrosine kinases, such as c-Src, c-Yes, and protein kinase C (PKC), have been found to be localized at these tight junctions, presumably indicating their pivotal role in the maintenance of tight junction integrity^54,55^. A significant body of evidence has highlighted the role of cytokines in the regulation of various tight junction proteins in a multitude of pathological conditions.

Tumor necrosis factor-α (TNFα), interferon-γ (IFN-γ), and interleukins all are well-known for their indisputable role in the regulation of tight junction integrity^56^. TNFα is a key player in the caveolin-1-mediated internalization of occludin, which elevates gut permeability; further, the overexpression of occludin alleviates the cytokine-induced increase in gut permeability^57^. TNFα stimulation of the NFκB signal transduction pathway is another major mechanism involved in tight junction regulation^58^. NFκB inhibition protected mice from severe water loss and diarrhea, which indicated its role in the regulation of the barrier property of IECs. The mechanism through which IFNγ modulates epithelial permeability is still under investigation; however, the acto-myosin cytoskeletal interaction with tight junction proteins is thought to be altered by IFNγ treatment^59–61^. IFNγ also induces an increase in barrier permeability through the reduction of ZO-1 and occludin expression in an adenosine monophosphate-activated protein kinase (AMPK)-dependent pathway, irrespective of the cellular energy levels^62^. The simultaneous presence of both these cytokines has a detrimental effect on intestinal integrity through the disassociation of tight junction proteins^63,64^.

A prominent player in cytokine-mediated tight junction regulation is myosin light chain kinase (MLCK)^56,65,66^, which disrupts the interaction between the tight junction proteins and the actin-myosin cytoskeleton, subsequently damaging the tight junction scaffold, which is crucial for the maintenance of barrier integrity^66–68^. TNF-mediated endocytosis of the tight junction requires enhanced MLCK transcription and activity at the tight junctions. Cytokines are also responsible for occludin redistribution from the tight junction to caveolin-containing vesicles^69^, and MLCK is also involved in the regulation of tight junction proteins through the alteration of ZO-1 protein dynamics^70^.

Another major mechanism of tight junction regulation is post-translational phosphorylation, which drastically alters their membrane distribution and turnover. Among the tight junction proteins, the post-translational phosphorylation of occludin has been widely studied and is responsible for opening and sealing tight junctions^71^. The phosphorylation status of occludin is intricately regulated by several kinases. Various research groups have suggested that serine/threonine phosphorylation is the predominant phosphorylation modification of occludin; however, recent research advances in this area have emphasized the importance of tyrosine phosphorylation. Hence, kinases such as PKC and c-Src and phosphatases, including PP2A, PP1, and PTP1B, which phosphorylate and dephosphorylate occludin, have a crucial role to play in intestinal barrier integrity^72–74^.

Occludin is highly phosphorylated at serine and threonine residues in the basal epithelium^72^. The specific kinases involved in occludin phosphorylation remain elusive; however, cellular/tight junction localization studies have suggested PKC as one of the cardinal players. Phosphorylated occludin interacts with ZO-1 and other tight junction proteins. An alteration in the phosphorylation pattern, such as an increase in tyrosine phosphorylation, which results from pathological conditions, such as inflammation or elevated ROS, can alter the protein–protein interactions of occludin with ZO-1, ZO-2, and ZO-3, and thereby alter the membrane integrity^75^. Oxidative-stress-induced intestinal permeability is thought to be mediated through the tyrosine phosphorylation of occludin and the redistribution of occludin, ZO-1, E-cadherin, and β-catenin from the intracellular junctions^76,77^. The tyrosine kinases involved in occludin phosphorylation remain largely obscure, although certain studies have speculated the role of c-Src family kinases in hydrogen peroxide-induced occludin tyrosine phosphorylation at the cellular level^31,78^. Tyrosine phosphorylated occludin could delocalize/disassemble from the tight junctions and undergo proteasome-mediated degradation, a phenomenon observed in patients suffering from inflammatory bowel syndrome^79,80^. The cellular energy sensor, AMPK, has been implicated in tight junction assembly by several studies^81^. AMPK is activated during the calcium switch-induced assembly of ZO-1, which facilitates tight junction assembly^81^. Butyrate, a short chain fatty acid abundant in the gut after the bacterial fermentation of carbohydrates, also induces tight junction assembly of ZO-1 and occludin through an AMPK-dependent pathway^82^. Although studies in this field have elucidated some of the mechanisms of tight junction regulation, in vivo studies that describe their role in pathological conditions are lacking. Despite the discoveries identifying the indisputable role of the post-translational phosphorylation of tight junction proteins, future studies to decipher the kinases and phosphatases involved, in addition to the putative binding partners and protein–protein interactions, are imperative for a better understanding of their regulatory mechanisms.

## Regulation of barrier integrity during pathological conditions

**Fig. 2:**
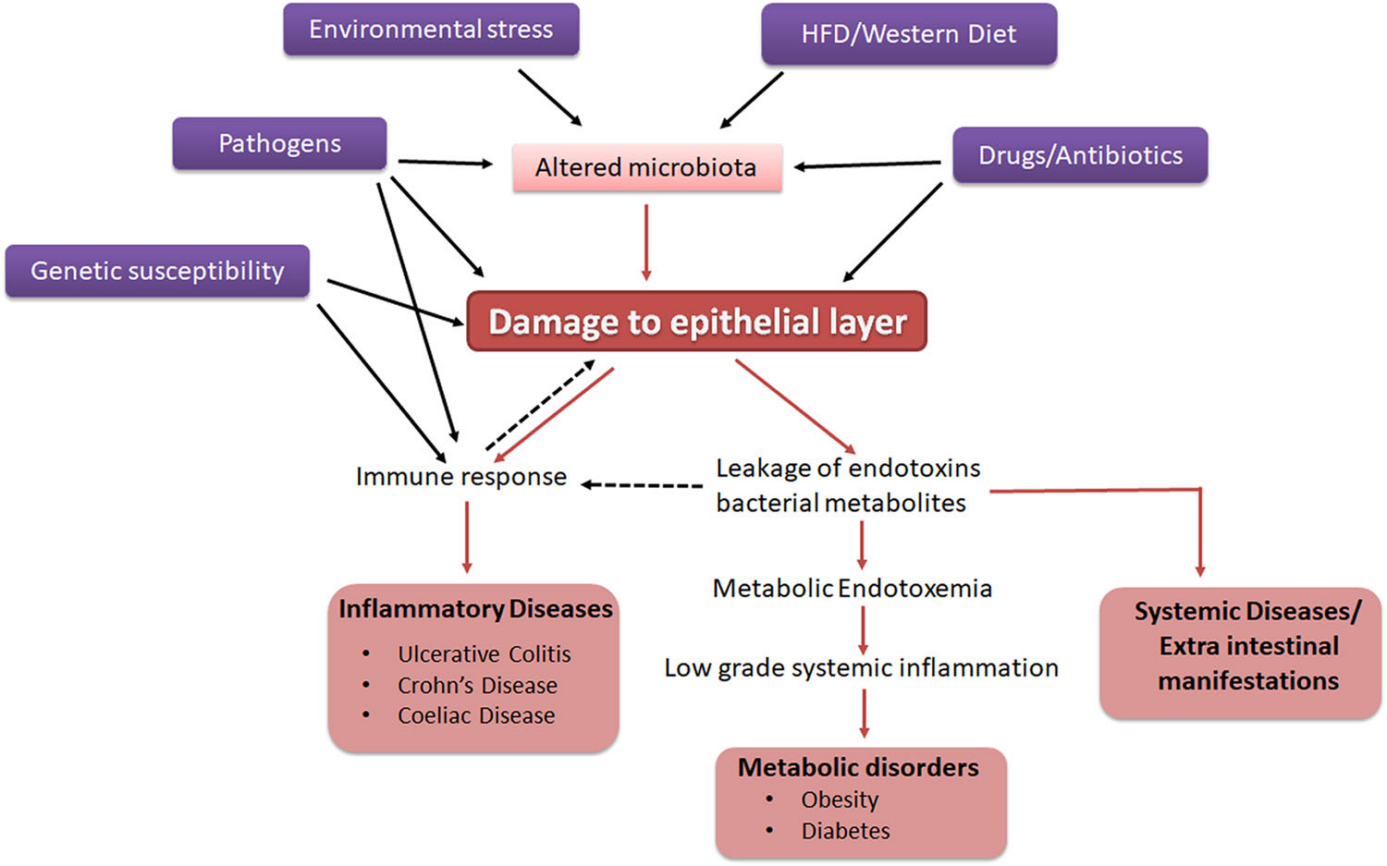
Factors affecting intestinal barrier integrity and pathological implications. Genetic susceptibility, diet, and a number of environmental conditions affect barrier integrity directly or indirectly through changes in microbiota. Compromised barrier integrity leads to an immune response and contributes to several pathological conditions

The significance of the gut barrier in disease pathogenesis has recently attracted attention. Compromised intestinal barrier integrity is observed in both intestinal and systemic diseases, including IBDs, autoimmune diseases, and other metabolic diseases^83^. However, the scientific community has not yet determined whether the loss of barrier integrity is the cause or consequence of these diseases. Pathophysiological or environmental factors may be the crucial factors that usurp normal physiology and increase the permeability of the barrier. Hence, it is imperative to understand the factors that contribute to the loss of barrier integrity under pathological conditions (Fig. 2).

## Inflammatory bowel disease and ulcerative colitis

IBD is a group of conditions in which patients have severe inflammation in the gastrointestinal tract^84–86^. It is a chronic condition that affects one out of 250 people in the European population as well as a significant population of people of other ethnic origins, and it has an unknown etiology, poor prognosis, and lifelong morbidity in patients^87,88^. There are two major classifications of IBD: ulcerative colitis (UC) and Crohn’s disease (CD). In UC, the inflammation is restricted to the rectum and the colon and never affects the small intestine; in contrast, in CD, there can be severe inflammation in any part of the intestine, including the small intestine and the large intestine^89^. The mechanism and etiology of disease progression are entirely different for these two types of IBD. Genetic predisposition to the disease is observed widely; in most cases, patients have at least one or more members in the family with the disease. Although the exact cause and mechanism of both types of IBD are yet to be completely understood, environmental or autoimmune-related factors are major causative factors^90,91^. The dysregulation of the mucosal immune response is also thought to be primarily responsible for disease progression^92,93^.

The loss of barrier integrity is a characteristic feature of IBD. A leaky gut may be an initial event in the pathogenesis of inflammatory bowel disorders, allowing bacteria-derived molecules into the mucosa and flaring up uncontrollable inflammatory signal cascades. The altered expression of tight junction proteins is observed in patients with UC and IBD^94^. Although there is an unresolved dispute over the contribution of the tight junction barrier to the disease pathology, recent studies have concluded that even in the presence of a normal underlying immunity, functional abnormalities of tight junction proteins could result in these diseases. In patients with CD, an increase in epithelial permeability precedes disease relapse, which emphasizes the essential role of tight junction proteins^95,96^. It has been reported that first-degree relatives of patients with IBD have abnormal intestinal permeability^97^, but it is still unclear whether individuals are genetically pre-disposed to compromised barrier integrity or if diet or environmental factors make them susceptible to the disease. The increase in apoptosis in IECs and cell shedding could also be responsible for the leakiness observed in these patients. However, some genetic studies have revealed a potential link between mutations in TJ-associated proteins, such as myosin IXB (MYO9B), partitioning defect protein (PARD3), PDZ containing protein 2 gene (MAGI2), and the development of IBD and celiac diseases^98–100^. All these studies reinforce the important role played by tight junction proteins and highlight the central role of epithelial barrier function in the pathogenesis of IBD.

Studies on in vivo experimental and spontaneous colitis models have identified the quintessential role played by tight junction proteins in the pathogenesis of UC. The roles of several kinases and proteins in the regulation of tight junction protein expression in pathological conditions were also investigated in these studies. Recently, it was reported in an experimental colitis model that dextran sodium sulfate (DSS) treatment, a chemical agent known to induce colitis, elevated the c-Src-mediated tyrosine phosphorylation of occludin in a phospholipase D2 (PLD2)-mediated pathway in PLD2 knockout mice. The inhibition of this pathway was shown to ameliorate DSS-induced colitis^101^.

## Obesity and metabolic disorders

The loss of barrier integrity is closely associated with the onset of metabolic disorders, including obesity and type-II diabetes (T2D). Clinical studies have shown that increased intestinal permeability decreased to within the normal range after weight reduction in patients with obesity^102^. Recent studies have also demonstrated a difference in the intestinal permeability between individuals with or without T2D, which implicated a crucial contribution of intestinal permeability to metabolic disorders^103^. However, further studies are necessary before the regulation of barrier permeability can be introduced into clinical practice^104^.

Metabolic endotoxemia, arising from the loss of barrier integrity, is thought to be a major factor that contributes to insulin resistance and obesity^105–107^. Damage to the intestinal epithelial layer causes the leakage of gut microbiota-derived lipopolysaccharide (LPS) and other toxins into the blood stream, resulting in metabolic endotoxemia. Mice fed a high-fat diet for 4 weeks showed a three- to fourfold increase in serum LPS, which is defined as metabolic endotoxemia. This condition can subsequently lead to low-grade systemic inflammation and insulin resistance that is central to metabolic diseases. LPS itself is known to impact the increase in gut permeability. Physiologically relevant LPS concentrations (0–10 ng/ml) can induce intestinal tight junction permeability in enterocytes via an increase in TLR-4 and CD14 expression, without inducing cell death^108^. As bacterial-derived LPS has a key role in the increase in gut permeability, changes in the gut microbiota must be a contributory factor to the pathogenesis of obesity and diabetes^109^.

Changes in the gut microbial composition are a characteristic feature of many metabolic diseases; obese and diabetic individuals show drastic differences in their gut microbiomes compared with healthy counterparts, and the gut microbiome plays a pivotal role in the maintenance of barrier integrity^110–112^. The two major bacterial phyla in the gut, Bacteriodetes and Firmicutes, have been investigated widely for their effect on IECs. Mice fed with an high fat diet (HFD), or patients with obesity or diabetes show a change in this ratio with elevated levels of Firmicutes and Proteobacteria compared with the beneficial species, Bacteriodetes. *Akkermansia muciniphila* is one such beneficial bacterial species known to be less abundant in obese and diabetic individuals and has been implicated in the regulation of intestinal barrier integrity^113^. The daily administration of live *A. muciniphila* has been shown to mitigate HFD-induced gut barrier dysfunction^114,115^. Bacterial-derived components have also been reported to directly affect tight junction assembly. Plovier et al.^116^ recently identified that a specific outer membrane protein of *A. muciniphila*, termed Amuc100, improved the gut barrier integrity and that administration of this protein alone could partly recapitulate the beneficial effects shown by *A. muciniphila*. Extracellular vesicles (EVs) secreted from bacteria are other major components that are known to interact directly with IECs and exert their function. EVs from *A. muciniphila* have been shown to improve the symptoms of DSS-induced colitis in mice^117^. Another study from our group recently showed that EVs derived from *A. muciniphila* improved barrier integrity and glucose tolerance and reduced body weight gain in HFD-fed mice (in press). The reduction in metabolic endotoxemia through the improvement of gut barrier permeability could therefore present an interesting strategy to treat metabolic disorders such as obesity and diabetes.

## Future perspectives

Recent studies have provided substantial evidence for the role of intestinal permeability in the regulation of several intestinal and extra-intestinal diseases. The improvement of barrier integrity by the regulation of tight junction protein expression or through other mechanisms has shown promising results with improvement of the disease symptoms in UC, CD, and metabolic diseases; strategies to identify and develop novel therapeutic targets to improve gut barrier integrity have become increasingly more attractive. The improvement of gut barrier integrity alone might not be sufficient in severe inflammatory diseases. However, in combination with conventional immunosuppressant drugs, such as TNFα inhibitors, approaches to improve intestinal barrier might prove beneficial. Metagenomic studies on gut microbiota from individuals with various diseases have shown that gut microbiota also actively interact with IECs for the regulation of barrier integrity. Various research groups are also investigating the strategy of probiotic and prebiotic administration to improve intestinal barrier integrity, as well as metabolic diseases. However, therapeutic intervention for the regulation of barrier integrity is an emerging topic and more investigations are essential to understand the role of intestinal barrier integrity in various diseases. The elucidation of signaling pathways involved in the regulation of the tight junction would allow the identification of novel barrier-restoring agents, which is imperative for deciphering novel and potent approaches for disease treatment.
